# Classical Presentation of Acute Appendicitis in the Case of a Subhepatic Appendix

**DOI:** 10.7759/cureus.6035

**Published:** 2019-10-30

**Authors:** Simran K Longani, Ahmed Ahmed

**Affiliations:** 1 Medicine, Imperial College London, London, GBR; 2 Upper Gastrointestinal and Bariatric Surgery, ‎Imperial College Healthcare National Health Service Trust, London, GBR

**Keywords:** appendicitis, subhepatic appendix, malrotated caecal pole, adhesions

## Abstract

Acute appendicitis is a very common surgical emergency diagnosed by combining the history, examination, and investigations to build a clinical picture. This presentation can become more complex with abnormal anatomical variations of the appendix. This case describes the rare clinical finding of a subhepatically located appendix and caecum in a 24-year-old female presenting with right lower quadrant (RLQ) pain. Examination findings were consistent with acute appendicitis. Ultrasonography identified the appendix as being located in the subhepatic region with laparoscopy confirming this finding and the presence of a malrotated caecal pole due to congenital adhesions. Laparoscopic appendectomy was subsequently performed therapeutically with no complications. This case focuses on the typical presentation of appendicitis and RLQ pain in a patient with an atypical anatomical structure. It aims to depict the importance of a widened knowledge of the aberrantly located appendix and how this can impact clinical presentation.

## Introduction

Appendicitis is one of the most common causes of abdominal pain in the younger population and accounts for over 40,000 hospital admissions per year across England. Classic symptoms include right iliac fossa (RIF) pain, anorexia, nausea, constipation, and vomiting; however, these classical presentations only occur in 50% of people [1]. In the presence of an anatomical variant where the appendix is aberrantly located, as in this case study of a subhepatic appendix, the clinical picture can be skewed making the initial diagnosis of appendicitis more complex.

## Case presentation

History

JN is a 24-year-old female who presented to the accident & emergency department (A&E) with a four-hour history of right lower quadrant (RLQ) abdominal pain. The pain originated in the umbilical region, radiating diffusely across the lower abdomen and subsequently localised to the RLQ. The pain was of sudden onset, sharp and colicky with progressing intensity. Over the counter, oral co-codamol 500mg (a combination analgesic of codeine phosphate and acetaminophen) was taken before presenting to A&E, which did not alleviate the pain. The pain was exacerbated by lifting the right leg and relieved by leaning forwards. Severity was rated eight on a scale of one to 10, with one being no pain and 10 being the most pain possible. This episode had not been preceded by previous abdominal pain, and she denied nausea or vomiting. She opened her bowels post-onset of the pain with no changes to the consistency of the stools and absence of blood or mucus. She denied urinary or infective symptoms. Past medical and surgical history was nil of note. Drug history included the oral contraceptive pill with no known drug allergies. There was no relevant family history. The patient did not smoke, reported alcohol consumption occasionally, and denied recreational drug use.

Examination

Under observation, JN was apyrexial with stable vital signs. The abdominal examination revealed a soft abdomen, tenderness on percussion, rebound tenderness in the RIF, and a positive psoas sign. She was not peritonitic and had a negative Rosving's sign and absent hernias.

Investigations

**Figure 1:**
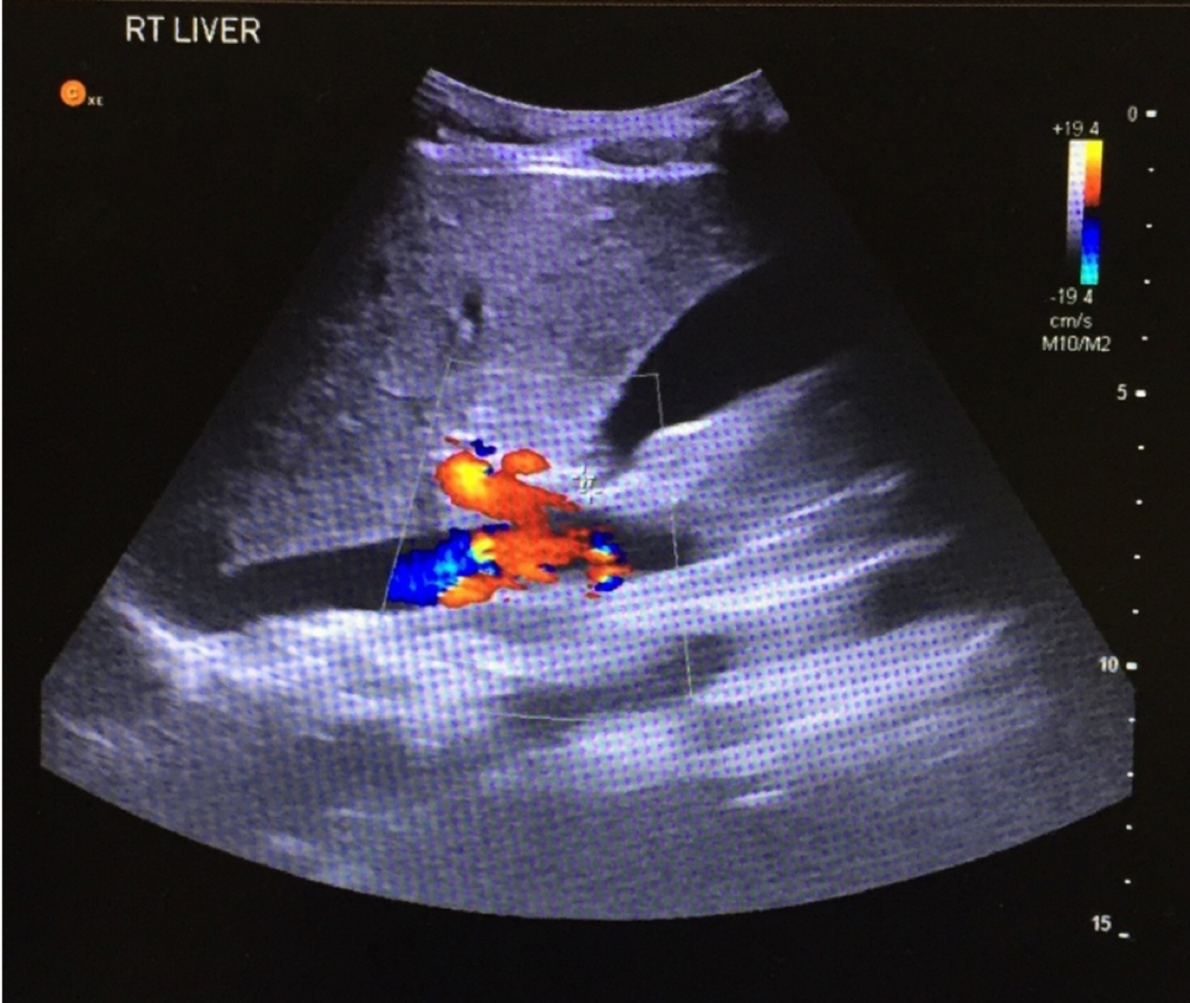
Longitudinal ultrasound visualising appendix at the level of the liver. Uncompressible appendix containing free fluid, measuring 6mm in diameter.

Based on the clinical presentation of JN, the initial impression pointed towards a provisional diagnosis of acute appendicitis, with ovarian cyst as a differential. Subsequent investigations revealed a negative urine dip and negative pregnancy test, which deemed a gynaecological cause unlikely. Blood results were all within normal ranges. Abdominal ultrasonography confirmed a diagnosis of appendicitis by the presence of free fluid within the RIF and within the 6mm appendix which was incompressible. These findings were in keeping with appendicitis. A key point to note is that the location of the appendix was a variant of the anatomical norm. It was visualised at the level of the right liver, indicating a subhepatically located appendix (Figure 1). This finding revised the diagnosis to subhepatic appendicitis.

Drugs

JN was commenced on the following medications whilst in A&E: Electrolyte solution infusion 1000ml IV, Paracetamol 1g oral, Morphine 10mg oral, Metronidazole 500mg IV. Following this, she was transferred to theatres for surgery.

Surgical report

A laparoscopic appendicectomy was performed under general anaesthesia with the following ports: umbilical 12mm, left iliac fossa 5mm, suprapubic 5mm. Reporting on the operation, the small bowel was dilated from the terminal ileum to proximal jejunum. The caecum was lying at the hepatic flexure and resultantly the appendix base was posteroinferior to the inferior border of the liver. This was attributed to a congenital adhesion causing malrotation of the caecal pole. The appendix itself did not appear inflamed or perforated and there was no free pus. Serous fluid was present in the pelvis and right upper quadrant (RUQ). Other than these observations, the sigmoid colon, uterus, ovaries and fallopian tubes were normal.

Upon discovering the anatomical variant, the adhesions were divided, caecum returned to the appropriate position in the RIF and appendix mobilised. There was diathermy and division of the appendicular artery. Vicryl endo-loops were applied, the appendix was divided and removed via the umbilical port. Finally, washout to the RIF and pelvis was performed and aspirated.

Following surgery, JN was stable and recovered well with mild bloating and tenderness. The patient was discharged with no prescription medication or follow up required.

## Discussion

It is rare for the caecum and appendix to be located subhepatically since subhepatic appendicitis makes up an annual incidence of 0.09 per 100,000 population [2]. In this patient, malrotation of the caecal pole resulted in the caecum being located at the hepatic flexure and appendix base posteroinferior to the inferior border of the liver. The cause, in this case, was attributed to congenital adhesions which tend to arise during physiological organogenesis or as a result of an abnormality in the embryonal development of the abdominal cavity [3].

The most reliable examination findings for acute appendicitis have been noted as tenderness on percussion, guarding, and rebound tenderness at the RIF [1]. When considering the anatomically higher appendix, it is noteworthy that examination findings are consistent with an appendix lying in the standard RIF location. Similar cases of subhepatic appendicitis accounted by Hafiz et al. (2017) and Ball et al. (2013) both report predominant RUQ pain as opposed to RLQ pain prompting an inaccurate consideration of biliary pathology [4,5]. In the case observed by Hafiz, a positive Murphy’s sign was also elicited despite previous cholecystectomy [4].

In many circumstances, atypical presentations of appendicitis are investigated using diagnostic laparoscopy. In this case, the initial intention of utilising a therapeutic approach of laparoscopic appendectomy reverted to diagnostic investigation upon discovery of the congenital adhesions and anatomical variation. Thus, laparoscopy proves beneficial for both diagnostic and therapeutic purposes in the case of anatomical variants.

## Conclusions

To conclude, this was a 24-year-old female who presented with RLQ pain and was diagnosed with acute appendicitis. The patient had an appendix located posteroinferior to the inferior border of the liver; laparoscopy revealed congenital adhesions causing malrotation of the caecal pole. Therapeutic laparoscopic appendectomy was performed with no complications. This case raises awareness of a rare case of subhepatic appendicitis where the pain was localised to the RLQ, but the anatomical position of the appendix was in the RUQ. It is important for clinicians to be aware of such anatomical variants and the use of laparoscopic appendectomy as both an exploratory and therapeutic technique.
